# *N*-(4-Chloro­phen­yl)-2-(naphthalen-1-yl)acetamide

**DOI:** 10.1107/S1600536812031613

**Published:** 2012-07-18

**Authors:** Hoong-Kun Fun, Ching Kheng Quah, Prakash S. Nayak, B. Narayana, B. K. Sarojini

**Affiliations:** a X-ray Crystallography Unit, School of Physics, Universiti Sains Malaysia, 11800 USM, Penang, Malaysia; b Department of Studies in Chemistry, Mangalore University, Mangalagangotri 574 199, India; c Department of Chemistry, P. A. College of Engineering, Nadupadavu, Mangalore 574 153, India

## Abstract

In the title compound, C_18_H_14_ClNO, the naphthalene ring system [maximum deviation = 0.014 (9) Å] forms a dihedral angle of 74.8 (2)° with the benzene ring. In the crystal, mol­ecules are linked *via* N—H⋯O hydrogen bonds into chains propagating along [010].

## Related literature

For general background to and related structures of the title compound, see: Fun *et al.* (2010 ▶, 2011*a* ▶,*b* ▶, 2012 ▶).
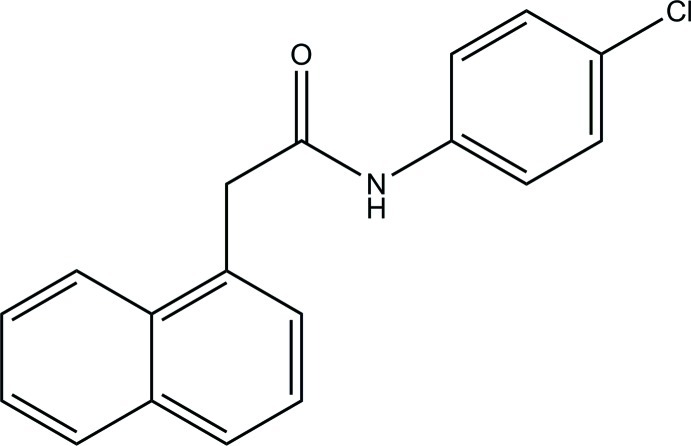


## Experimental

### 

#### Crystal data

C_18_H_14_ClNO
*M* *_r_* = 295.75
Monoclinic, 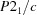

*a* = 19.163 (6) Å
*b* = 5.0458 (11) Å
*c* = 17.252 (4) Å
β = 116.365 (5)°
*V* = 1494.6 (7) Å^3^
*Z* = 4
Mo *K*α radiation
μ = 0.25 mm^−1^
*T* = 296 K
0.35 × 0.15 × 0.09 mm

#### Data collection

Bruker SMART APEXII DUO CCD diffractometer
Absorption correction: multi-scan (*SADABS*; Bruker, 2009 ▶) *T*_min_ = 0.916, *T*_max_ = 0.978
9206 measured reflections
2611 independent reflections
1185 reflections with *I* > 2σ(*I*)
*R*_int_ = 0.050

#### Refinement

*R*[*F* ^2^ > 2σ(*F* ^2^)] = 0.060
*wR*(*F* ^2^) = 0.208
*S* = 1.01
2611 reflections
195 parameters
H atoms treated by a mixture of independent and constrained refinement
Δρ_max_ = 0.32 e Å^−3^
Δρ_min_ = −0.18 e Å^−3^

### 

Data collection: *APEX2* (Bruker, 2009 ▶); cell refinement: *SAINT* (Bruker, 2009 ▶); data reduction: *SAINT*; program(s) used to solve structure: *SHELXTL* (Sheldrick, 2008 ▶); program(s) used to refine structure: *SHELXTL*; molecular graphics: *SHELXTL*; software used to prepare material for publication: *SHELXTL* and *PLATON* (Spek, 2009 ▶).

## Supplementary Material

Crystal structure: contains datablock(s) global, I. DOI: 10.1107/S1600536812031613/hb6893sup1.cif

Structure factors: contains datablock(s) I. DOI: 10.1107/S1600536812031613/hb6893Isup2.hkl

Supplementary material file. DOI: 10.1107/S1600536812031613/hb6893Isup3.cml

Additional supplementary materials:  crystallographic information; 3D view; checkCIF report

## Figures and Tables

**Table 1:** Hydrogen-bond geometry (Å, °)

*D*—H⋯*A*	*D*—H	H⋯*A*	*D*⋯*A*	*D*—H⋯*A*
N1—H1N1⋯O1^i^	0.98 (5)	1.98 (5)	2.942 (4)	166 (4)

Symmetry code: (i) 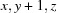.

**Fig. 1.**
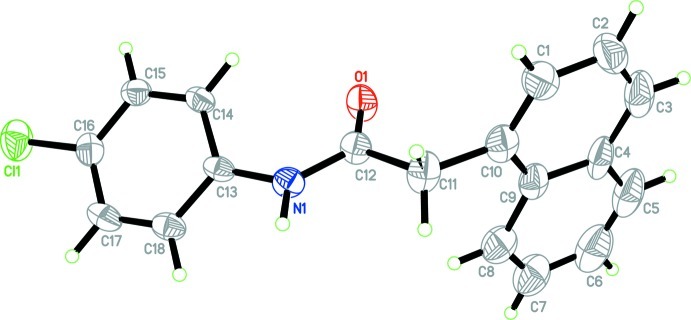
The molecular structure of the title compound showing 50% probability displacement ellipsoids for non-H atoms.

**Fig. 2.**
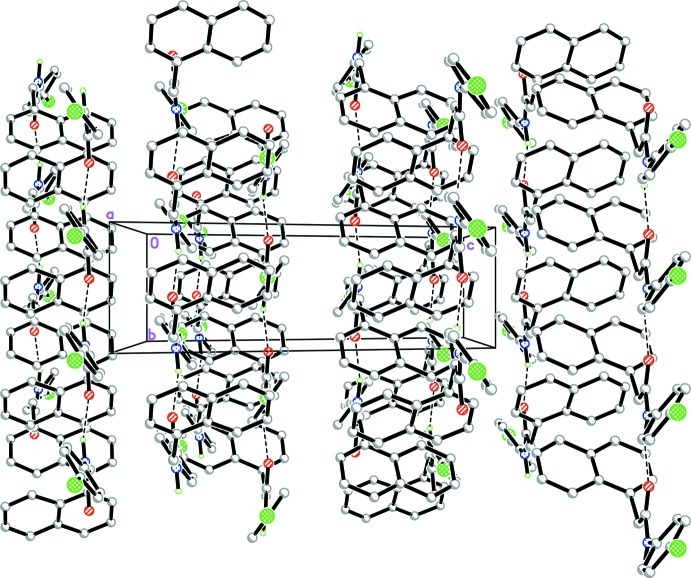
The crystal structure of the title compound, viewed along the a axis. H atoms not involved in hydrogen bonds (dashed lines) have been omitted for clarity.

## supplementary crystallographic information

### Comment

In continuation of our work on synthesis of amides (Fun *et al.*, 2010, 2011*a*,*b*, 2012), we report herein the crystal structure of the title compound.

The molecular structure is shown in Fig. 1. Bond lengths are comparable to related structures (Fun *et al.*, 2010, 2011*a*,*b*, 2012). The naphthalene ring system (C1–C10, maximum deviation of 0.014 (9) Å at atom C5) forms a dihedral angle of 74.8 (2)° with the benzene ring (C13–C18).

In the crystal structure, Fig. 2, molecules are linked *via* N1—H1N1···O1 hydrogen bonds (Table 1) into one-dimensional chains along [010].

### Experimental

1-Naphthalene acetic acid (0.186 g, 1 mmol), 4-chloroaniline (0.127 g, 1 mmol) and 1-ethyl-3-(3-dimethylaminopropyl)-carbodiimide hydrochloride (1.0 g, 0.01 mol) were dissolved in dichloromethane (20 ml). The mixture was stirred in presence of triethylamine at 273 K for about 3 h. The contents were poured into 100 ml of ice-cold aqueous hydrochloric acid with stirring, which was extracted thrice with dichloromethane. Organic layer was washed with saturated NaHCO_3_ solution and brine solution, dried and concentrated under reduced pressure to give the title compound. Colourless blocks were grown from acetone and toluene (1:1) mixture by the slow evaporation method (m.p. 449–451 K).

### Refinement

Atom H1N1 was located in a difference Fourier map and refined freely with N1—H1N1 = 0.98 (5) Å. The remaining H atoms were positioned geometrically and refined using a riding model with C—H = 0.93 or 0.97 Å and *U*_iso_(H) = 1.2*U*_eq_(C).

### Crystal data

**Table:**

C_18_H_14_ClNO	*F*(000) = 616
*M**_r_* = 295.75	*D*_x_ = 1.314 Mg m^−^^3^
Monoclinic, *P*2_1_/*c*	Mo *K*α radiation, λ = 0.71073 Å
Hall symbol: -P 2ybc	Cell parameters from 1386 reflections
*a* = 19.163 (6) Å	θ = 2.4–21.5°
*b* = 5.0458 (11) Å	µ = 0.25 mm^−^^1^
*c* = 17.252 (4) Å	*T* = 296 K
β = 116.365 (5)°	Block, colourless
*V* = 1494.6 (7) Å^3^	0.35 × 0.15 × 0.09 mm
*Z* = 4	

### Data collection

**Table:**

Bruker SMART APEXII DUO CCD diffractometer	2611 independent reflections
Radiation source: fine-focus sealed tube	1185 reflections with *I* > 2σ(*I*)
Graphite monochromator	*R*_int_ = 0.050
φ and ω scans	θ_max_ = 25.0°, θ_min_ = 2.4°
Absorption correction: multi-scan (*SADABS*; Bruker, 2009)	*h* = −22→22
*T*_min_ = 0.916, *T*_max_ = 0.978	*k* = −5→3
9206 measured reflections	*l* = −20→19

### Refinement

**Table:**

Refinement on *F*^2^	Secondary atom site location: difference Fourier map
Least-squares matrix: full	Hydrogen site location: inferred from neighbouring sites
*R*[*F*^2^ > 2σ(*F*^2^)] = 0.060	H atoms treated by a mixture of independent and constrained refinement
*wR*(*F*^2^) = 0.208	*w* = 1/[σ^2^(*F*_o_^2^) + (0.0913*P*)^2^ + 0.5172*P*] where *P* = (*F*_o_^2^ + 2*F*_c_^2^)/3
*S* = 1.01	(Δ/σ)_max_ = 0.001
2611 reflections	Δρ_max_ = 0.32 e Å^−^^3^
195 parameters	Δρ_min_ = −0.18 e Å^−^^3^
0 restraints	Extinction correction: *SHELXTL* (Sheldrick, 2008), Fc^*^=kFc[1+0.001xFc^2^λ^3^/sin(2θ)]^-1/4^
Primary atom site location: structure-invariant direct methods	Extinction coefficient: 0.008 (3)

### Special details

**Table:**

Geometry. All esds (except the esd in the dihedral angle between two l.s. planes) are estimated using the full covariance matrix. The cell esds are taken into account individually in the estimation of esds in distances, angles and torsion angles; correlations between esds in cell parameters are only used when they are defined by crystal symmetry. An approximate (isotropic) treatment of cell esds is used for estimating esds involving l.s. planes.
Refinement. Refinement of F^2^ against ALL reflections. The weighted R-factor wR and goodness of fit S are based on F^2^, conventional R-factors R are based on F, with F set to zero for negative F^2^. The threshold expression of F^2^ > 2sigma(F^2^) is used only for calculating R-factors(gt) etc. and is not relevant to the choice of reflections for refinement. R-factors based on F^2^ are statistically about twice as large as those based on F, and R-factors based on ALL data will be even larger.

### Fractional atomic coordinates and isotropic or equivalent isotropic displacement parameters (Å^2^)

**Table:**

	*x*	*y*	*z*	*U*_iso_*/*U*_eq_	
Cl1	0.30184 (7)	0.9142 (3)	0.11979 (9)	0.1183 (7)	
O1	0.64245 (16)	0.5769 (5)	0.1247 (2)	0.0885 (10)	
N1	0.6142 (2)	1.0067 (7)	0.1325 (2)	0.0681 (10)	
C1	0.8073 (3)	0.5845 (12)	0.0810 (4)	0.1051 (16)	
H1A	0.7783	0.6565	0.0263	0.126*	
C2	0.8600 (4)	0.3825 (15)	0.0897 (6)	0.130 (2)	
H2A	0.8650	0.3194	0.0418	0.156*	
C3	0.9041 (4)	0.2791 (13)	0.1703 (7)	0.138 (3)	
H3A	0.9402	0.1460	0.1780	0.166*	
C4	0.8942 (3)	0.3771 (11)	0.2422 (5)	0.1032 (17)	
C5	0.9390 (4)	0.2649 (14)	0.3251 (8)	0.147 (3)	
H5A	0.9739	0.1276	0.3328	0.176*	
C6	0.9296 (5)	0.363 (2)	0.3916 (7)	0.166 (3)	
H6A	0.9598	0.2925	0.4463	0.199*	
C8	0.8326 (3)	0.6693 (12)	0.3047 (5)	0.1102 (17)	
H8A	0.7972	0.8025	0.2994	0.132*	
C7	0.8779 (4)	0.5606 (17)	0.3840 (5)	0.133 (2)	
H7A	0.8737	0.6209	0.4326	0.160*	
C9	0.8398 (3)	0.5767 (9)	0.2290 (4)	0.0863 (14)	
C10	0.7961 (3)	0.6808 (9)	0.1472 (4)	0.0880 (14)	
C11	0.7373 (2)	0.9033 (9)	0.1340 (3)	0.0958 (14)	
H11A	0.7606	1.0297	0.1810	0.115*	
H11B	0.7266	0.9955	0.0806	0.115*	
C12	0.6607 (2)	0.8082 (8)	0.1304 (3)	0.0723 (11)	
C13	0.5384 (2)	0.9812 (7)	0.1258 (2)	0.0589 (10)	
C14	0.4866 (2)	0.7824 (7)	0.0781 (2)	0.0666 (11)	
H14A	0.5013	0.6598	0.0479	0.080*	
C15	0.4140 (3)	0.7658 (8)	0.0751 (3)	0.0710 (11)	
H15A	0.3801	0.6312	0.0436	0.085*	
C16	0.3914 (2)	0.9466 (9)	0.1184 (3)	0.0748 (12)	
C17	0.4414 (3)	1.1482 (8)	0.1643 (3)	0.0794 (13)	
H17A	0.4259	1.2735	0.1931	0.095*	
C18	0.5136 (3)	1.1623 (8)	0.1672 (2)	0.0717 (11)	
H18B	0.5469	1.2989	0.1982	0.086*	
H1N1	0.630 (3)	1.193 (10)	0.140 (3)	0.115 (16)*	

### Atomic displacement parameters (Å^2^)

**Table:**

	*U*^11^	*U*^22^	*U*^33^	*U*^12^	*U*^13^	*U*^23^
Cl1	0.0979 (10)	0.1515 (14)	0.1228 (12)	0.0188 (9)	0.0646 (9)	0.0142 (10)
O1	0.0811 (19)	0.0456 (17)	0.144 (3)	−0.0048 (15)	0.0547 (19)	−0.0001 (17)
N1	0.077 (2)	0.0456 (19)	0.080 (2)	−0.0074 (18)	0.0337 (19)	0.0019 (17)
C1	0.102 (4)	0.106 (4)	0.128 (5)	−0.025 (3)	0.069 (4)	−0.014 (4)
C2	0.111 (5)	0.121 (5)	0.196 (7)	−0.027 (4)	0.102 (5)	−0.054 (5)
C3	0.083 (4)	0.085 (4)	0.261 (10)	−0.017 (3)	0.090 (6)	−0.022 (6)
C4	0.052 (3)	0.076 (3)	0.175 (6)	−0.002 (3)	0.045 (4)	0.020 (4)
C5	0.077 (4)	0.095 (5)	0.247 (10)	−0.004 (3)	0.053 (6)	0.030 (6)
C6	0.105 (6)	0.170 (9)	0.208 (10)	−0.025 (6)	0.056 (6)	0.021 (8)
C8	0.094 (4)	0.114 (4)	0.128 (5)	−0.027 (3)	0.054 (4)	−0.001 (4)
C7	0.105 (5)	0.166 (7)	0.129 (6)	−0.022 (5)	0.052 (4)	0.011 (5)
C9	0.067 (3)	0.062 (3)	0.133 (5)	−0.015 (2)	0.048 (3)	0.001 (3)
C10	0.079 (3)	0.066 (3)	0.128 (5)	−0.019 (3)	0.054 (3)	0.003 (3)
C11	0.078 (3)	0.065 (3)	0.143 (4)	−0.006 (3)	0.049 (3)	0.020 (3)
C12	0.077 (3)	0.046 (2)	0.093 (3)	0.000 (2)	0.037 (2)	0.008 (2)
C13	0.087 (3)	0.040 (2)	0.052 (2)	0.004 (2)	0.032 (2)	0.0031 (17)
C14	0.080 (3)	0.051 (2)	0.070 (3)	0.003 (2)	0.035 (2)	−0.0098 (19)
C15	0.076 (3)	0.065 (3)	0.067 (3)	−0.005 (2)	0.026 (2)	−0.006 (2)
C16	0.077 (3)	0.092 (3)	0.064 (3)	0.016 (3)	0.039 (2)	0.015 (3)
C17	0.119 (4)	0.061 (3)	0.068 (3)	0.010 (3)	0.051 (3)	−0.003 (2)
C18	0.098 (3)	0.053 (2)	0.065 (3)	−0.002 (2)	0.038 (2)	−0.001 (2)

### Geometric parameters (Å, º)

**Table:**

Cl1—C16	1.736 (4)	C8—C9	1.451 (7)
O1—C12	1.210 (4)	C8—H8A	0.9300
N1—C12	1.352 (5)	C7—H7A	0.9300
N1—C13	1.411 (5)	C9—C10	1.386 (6)
N1—H1N1	0.98 (5)	C10—C11	1.535 (6)
C1—C10	1.344 (7)	C11—C12	1.518 (6)
C1—C2	1.394 (8)	C11—H11A	0.9700
C1—H1A	0.9300	C11—H11B	0.9700
C2—C3	1.371 (9)	C13—C18	1.368 (5)
C2—H2A	0.9300	C13—C14	1.395 (5)
C3—C4	1.423 (9)	C14—C15	1.371 (5)
C3—H3A	0.9300	C14—H14A	0.9300
C4—C9	1.394 (7)	C15—C16	1.365 (5)
C4—C5	1.420 (10)	C15—H15A	0.9300
C5—C6	1.331 (10)	C16—C17	1.380 (6)
C5—H5A	0.9300	C17—C18	1.365 (6)
C6—C7	1.372 (10)	C17—H17A	0.9300
C6—H6A	0.9300	C18—H18B	0.9300
C8—C7	1.367 (8)		
			
C12—N1—C13	126.7 (3)	C1—C10—C9	118.4 (5)
C12—N1—H1N1	123 (3)	C1—C10—C11	121.6 (6)
C13—N1—H1N1	111 (3)	C9—C10—C11	120.0 (5)
C10—C1—C2	123.6 (6)	C12—C11—C10	114.0 (3)
C10—C1—H1A	118.2	C12—C11—H11A	108.7
C2—C1—H1A	118.2	C10—C11—H11A	108.7
C3—C2—C1	118.6 (7)	C12—C11—H11B	108.7
C3—C2—H2A	120.7	C10—C11—H11B	108.7
C1—C2—H2A	120.7	H11A—C11—H11B	107.6
C2—C3—C4	119.4 (7)	O1—C12—N1	123.1 (4)
C2—C3—H3A	120.3	O1—C12—C11	123.3 (4)
C4—C3—H3A	120.3	N1—C12—C11	113.7 (4)
C9—C4—C5	121.8 (7)	C18—C13—C14	117.8 (4)
C9—C4—C3	119.2 (7)	C18—C13—N1	118.7 (4)
C5—C4—C3	119.0 (7)	C14—C13—N1	123.4 (3)
C6—C5—C4	117.9 (8)	C15—C14—C13	120.7 (4)
C6—C5—H5A	121.0	C15—C14—H14A	119.7
C4—C5—H5A	121.0	C13—C14—H14A	119.7
C5—C6—C7	123.7 (10)	C16—C15—C14	120.1 (4)
C5—C6—H6A	118.2	C16—C15—H15A	119.9
C7—C6—H6A	118.2	C14—C15—H15A	119.9
C7—C8—C9	119.9 (6)	C15—C16—C17	120.0 (4)
C7—C8—H8A	120.0	C15—C16—Cl1	120.0 (4)
C9—C8—H8A	120.0	C17—C16—Cl1	119.9 (4)
C8—C7—C6	120.0 (8)	C18—C17—C16	119.5 (4)
C8—C7—H7A	120.0	C18—C17—H17A	120.2
C6—C7—H7A	120.0	C16—C17—H17A	120.2
C10—C9—C4	120.7 (6)	C17—C18—C13	121.9 (4)
C10—C9—C8	122.6 (5)	C17—C18—H18B	119.1
C4—C9—C8	116.7 (6)	C13—C18—H18B	119.1
			
C10—C1—C2—C3	−1.4 (8)	C8—C9—C10—C11	0.0 (6)
C1—C2—C3—C4	0.9 (9)	C1—C10—C11—C12	101.4 (5)
C2—C3—C4—C9	0.0 (8)	C9—C10—C11—C12	−79.4 (5)
C2—C3—C4—C5	178.7 (5)	C13—N1—C12—O1	−2.2 (7)
C9—C4—C5—C6	−2.3 (9)	C13—N1—C12—C11	176.7 (4)
C3—C4—C5—C6	179.0 (6)	C10—C11—C12—O1	−10.9 (7)
C4—C5—C6—C7	1.6 (12)	C10—C11—C12—N1	170.2 (4)
C9—C8—C7—C6	−0.6 (8)	C12—N1—C13—C18	149.8 (4)
C5—C6—C7—C8	−0.2 (11)	C12—N1—C13—C14	−31.2 (6)
C5—C4—C9—C10	−179.2 (4)	C18—C13—C14—C15	−1.9 (5)
C3—C4—C9—C10	−0.5 (6)	N1—C13—C14—C15	179.0 (4)
C5—C4—C9—C8	1.6 (6)	C13—C14—C15—C16	0.8 (6)
C3—C4—C9—C8	−179.7 (4)	C14—C15—C16—C17	0.7 (6)
C7—C8—C9—C10	−179.3 (4)	C14—C15—C16—Cl1	−176.3 (3)
C7—C8—C9—C4	−0.2 (7)	C15—C16—C17—C18	−1.0 (6)
C2—C1—C10—C9	0.8 (7)	Cl1—C16—C17—C18	176.0 (3)
C2—C1—C10—C11	−179.9 (4)	C16—C17—C18—C13	−0.2 (6)
C4—C9—C10—C1	0.2 (6)	C14—C13—C18—C17	1.6 (5)
C8—C9—C10—C1	179.3 (4)	N1—C13—C18—C17	−179.3 (3)
C4—C9—C10—C11	−179.1 (4)		

### Hydrogen-bond geometry (Å, º)

**Table:**

*D*—H···*A*	*D*—H	H···*A*	*D*···*A*	*D*—H···*A*
N1—H1*N*1···O1^i^	0.98 (5)	1.98 (5)	2.942 (4)	166 (4)

Symmetry code: (i) *x*, *y*+1, *z*.
